# Survival of Patients with Primary Brain Tumors: Comparison of Two Statistical Approaches

**DOI:** 10.1371/journal.pone.0148733

**Published:** 2016-02-10

**Authors:** Iveta Selingerová, Hana Doleželová, Ivanka Horová, Stanislav Katina, Jiří Zelinka

**Affiliations:** 1 Department of Mathematics and Statistics, Faculty of Science, Masaryk University, Kotlářská 2, 61137, Brno, Czech Republic; 2 Masaryk Memorial Cancer Institute, Žlutý kopec 7, 65653 Brno, Czech Republic; The George Washington University, UNITED STATES

## Abstract

**Purpose:**

We reviewed the survival time for patients with primary brain tumors undergoing treatment with stereotactic radiation methods at the Masaryk Memorial Cancer Institute Brno. We also identified risk factors and characteristics, and described their influence on survival time.

**Methods:**

In summarizing survival data, there are two functions of principal interest, namely, the survival function and the hazard function. In practice, both of them can depend on some characteristics. We focused on nonparametric methods, propose a method based on kernel smoothing, and compared our estimates with the results of the Cox regression model. The hazard function is conditional to age and gross tumor volume and visualized as a color-coded surface. A multivariate Cox model was also designed.

**Results:**

There were 88 patients with primary brain cancer, treated with stereotactic radiation. The median survival of our patient cohort was 47.8 months. The estimate of the hazard function has two peaks (about 10 months and about 40 months). The survival time of patients was significantly different for various diagnoses (p≪0.001), KI (p = 0.047) and stereotactic methods (p = 0.033). Patients with a greater GTV had higher risk of death. The suitable threshold for GTV is 20 cm^3^. Younger patients with a survival time of about 50 months had a higher risk of death. In the multivariate Cox regression model, the selected variables were age, GTV, sex, diagnosis, KI, location, and some of their interactions.

**Conclusion:**

Kernel methods give us the possibility to evaluate continuous risk variables and based on the results offer risk-prone patients a different treatment, and can be useful for verifying assumptions of the Cox model or for finding thresholds of continuous variables.

## Introduction

Primary brain tumors represent about 1–2% of all malignant tumors. The number of new cases of brain tumors is about 800 people per year in the Czech population, with a slight predominance in males. The incidence in 2011 was 8.5 per 100 000 males and 7.8 per 100 000 females. The number of deaths was 7.5 per 100 000 males 6.6 per 100 000 females per year (see [1]).

In brain tumors, stereotactic radiation methods offer the possibility of the application of high doses of radiation to a small volume with a high dose gradient to surrounding tissues. These methods are mostly used in the treatment of brain lesions, especially—brain metastases. In primary brain tumors, such as gliomas, meningiomas, acoustic neuromas and pituitary tumors, as well as arteriovenous malformations, a treatment can be performed using stereotactic methods—stereotactic radiosurgery (SRS) and fractionated stereotactic radiotherapy (SRT). SRS uses a single application of radiation and reminds us of neurosurgery. In SRT, the total dose is divided into several fractions with higher single dose. Accelerated hypofractionation is mostly used. In comparison to conformal radiotherapy, these methods bring a benefit of lower risk of side effects ([2], [3], [4], [5]).

The mostly used endpoint in primary brain tumor is disease-specific survival (DSS) which is studied by survival analysis methods. DSS is an event observed over time which can be associated with some characteristics, e.g. age, gender, type of tumor.

When analyzing the brain tumor data, the following questions arise:

What is the probability of a patient surviving a certain period of time? What circumstances indicate the highest risk of death and when does that risk change most rapidly? Can survival time be affected by the patient’s characteristics?

Establishing the survival patterns of cancer patients is an important statistical problem. Based on those results, a prognosis can be determined or a method of treatment chosen. The process of statistical analysis is made more difficult by incomplete observation (censoring). It is necessary to carefully choose a statistical method with respect to its assumptions. Inadequate results can be obtained where assumptions of methods are violated. This is the reason why non-parametric methods are used for analyzing survival data. However, some of these methods also require assumptions. Kernel methods used in this article are more flexible in this respect and produce functions more useful for presentation. Based on the shape of the hazard function, it could be possible to divide patients to two or more groups with similar risk of death to ease the interpretation. It might be possible to find one or more thresholds in direction of each covariate.

In this paper, we analyzed brain tumor data as a demonstration of using the hazard function and kernel methods in medical research. Kernel estimates offer an effective identification of risk groups of patients and can be useful in the other areas of medicine or cancer research. Due to the flexibility of these methods, it is possible to analyze the various data set. Note, however, that it is necessary to be careful in an interpretation of results.

## Materials and Methods

### Patients

Patients with primary brain tumors were treated at the Masaryk Memorial Cancer Institute in Brno. The patients who underwent stereotactic irradiation were advised for treatment by a multi-branch Commission for brain tumors. They then underwent preparation for CT and MR examinations. Planning and contouring of the target volume and organs at risk were carried out at the Brain-Lab with the cooperation of radiologist, neurosurgeon and radiotherapist to design a system for stereotactic radiotherapy. The patients were irradiated on a linear accelerator with microcolimator system (X knife), photon beam with 6 MV energy. After treatment they were monitored at the Clinic of Radiation Oncology as well as the Neurological ward and at regular intervals were examined via magnetic resonance (see [6] for details).

The first patient was included into the study on July 30, 2004, the last on June 27, 2011. The study was concluded on May 5, 2012. Eighty eight patients were selected from a total of 100 medical records. Patients under 11 years or with supra- and infratentorial location, or with modified radiosurgery, or with gross tumor volume greater than 80 cm^3^ were excluded.

### Ethics Statement

Ethics approval for the study was granted by the Ethics Committee of the Masaryk Memorial Cancer Institute. All procedures performed in studies involving human participants were in accordance with the ethical standards of the institutional research committee and with the Declaration of Helsinki and its later amendments. The patients were informed of the treatment side effects and signed an informed consent.

### Characteristics of patients

The most important variables of interest were age (in years), gross tumor volume (GTV—in cm^3^), sex (female, male), diagnosis (meningioma, LG glioma, HG glioma and other diagnoses), tumor location (infratentioral or supratentorial), Karnofsky index (KI, greater than or equal to 80%, less than 80%) and stereotactic methods (SRS, SRT). The basic statistical characteristics of these variables are given in Tables 1 and 2.

**Table 1:** Absolute and relative frequencies of selected variables.

	Censored	Dead	Censored (%)	Dead (%)
**Sex**				
female	30	15	66.67	33.33
male	23	20	53.49	46.51
**Diagnosis**				
Meningioma	33	9	78.57	21.43
LG glioma	5	4	55.56	44.44
HG glioma	5	17	22.73	77.27
other	9	5	64.29	35.71
**Location**				
infratentorial	15	4	78.95	21.05
supratentorial	38	31	55.07	44.93
**Karnofsky index**				
KI≥ 80%	43	25	63.24	36.76
KI< 80%	10	10	50.00	50.00
**Stereotactic methods**				
SRS	18	5	78.26	21.74
SRT	35	30	53.85	46.15

**Table 2:** Basic statistical characteristics of age and gross tumor volume.

	Median	Mean	SD	Min	Max
Age (years)	56.00	55.82	16.27	17.00	85.00
GTV (cm^3^)	6.51	8.56	8.38	0.01	30.41

Meningioma, LG glioma, HG glioma were the most common diagnoses for patients who underwent a stereotactic treatment. Other diagnoses (cerebellopontine angle tumor, craniopharyngioma, pituitary adenoma, choroid plexus tumor and penetrating head and neck tumor) constituted only a small percentage and therefore were combined into one group. The tumor location can be useful in determining the type of tumor. In this study, the patients were adults, therefore, the location of tumor above the tentorium (supratentorial) prevailed. The location below the tentorium (infratentioral) is more frequent among children. The Karnofsky Index, which characterizes the overall patient’s condition, runs from 100% to 0%, where 100% is perfect health and 0% is death. KI greater or equal to 80% is for patients who are able to carry on normal activities and work. Due to outliers, GTV was winsorized at the 96th percentile (3 patients).

A previous radiotherapy to the whole brain had been applied in 32 patients (36.4%), thus using stereotactic radiotherapy was reirradiation. The median dose of prior radiotherapy was 56 Gy (range 40–70 Gy). 23 patients who underwent SRS had a median dose of 18 Gy (range 14–40 Gy). 65 patients were irradiated using SRT technology with a median dose of 28 Gy (12–58 Gy).

### Statistical methods

A survival process can be characterized by the survival function $\bar{F}$ and hazard function *λ*. The survival function $\bar{F}\left(t\right)$ is the probability that the survival time is greater than or equal to *t*. The hazard function reflects the instantaneous probability that an individual dies within the next time instant (i.e. the probability that individual dies at the time *t* conditional on he or she having survived to that time). Both functions, survival and hazard, complement each other and present different viewpoints to the data. The hazard function is the derivative of the survival function at a specific time point divided by the value of the survival function at that point multiplied by −1, i.e.

$$\begin{array}{c} \lambda \left(t\right)=-\frac{{\bar{F}}^{\prime }\left(t\right)}{\bar{F}\left(t\right)}. \end{array}$$

If an appropriate probability distribution of survival time *T* is known, then the related survival characteristics (survival and hazard functions) can be calculated precisely. However, in many cases the distribution is not known and then the estimations of these indices need to be available. Therefore, we focused on methods of nonparametric estimations, especially of kernel estimates of the survival and hazard functions. These methods are simple to understand and possess good statistical properties of estimates (see [7]).

Moreover, in biomedical application the points of the most rapid decrease of the hazard function are of great interest. These points can be also estimated using kernel estimations.

The basic idea of kernel estimations can be expressed as follows: the value of the unknown function at a time *t* can be estimated as a local weighted average of known observations in the neighborhood of *t*. The kernel (or the kernel function) *K*(⋅) plays the role of the weight and the bandwidth *h* determines the size of the neighborhood. The kernel is usually a function with properties of probability density. The kernel estimator can be expressed for ordered observed times *Y*_(*i*)_ and corresponding censoring indicators *δ*_(*i*)_ (*δ*_(*i*)_ = 1 indicating death and *δ*_(*i*)_ = 0 indicating censored data) as

$$\begin{array}{c} \hat{\lambda }\left(t\right)=\frac{1}{h}\sum_{i=1}^{n}K\left(\frac{t-Y_{\left(i\right)}}{h}\right)\frac{\delta_{\left(i\right)}}{n-i+1}. \end{array}$$

The kernel estimator of the survival function is obtained using the relationship to the hazard function:

$$\begin{array}{c} \hat{\bar{F}}\left(t\right)=e^{-\int_{0}^{t}\hat{\lambda }\left(u\right)du}. \end{array}$$

We use the well-known Kaplan—Meier estimator of the survival function (see [8]) for the comparison with the kernel estimate:

$$\begin{array}{c} {\hat{\bar{F}}}_{KM}\left(t\right)=\prod_{i:Y_{\left(i\right)}<t}{\left(\frac{n-i}{n-i+1}\right)}^{\delta_{\left(i\right)}}. \end{array}$$

It is evident that the patient’s survival time also depends on age, gross of tumor volume, etc. The above mentioned hazard function cannot capture this influence. Thus we proceed to a conditional hazard function.

This function gives the instantaneous failure rate, assuming that a patient survives until a certain time and provided that some other characteristics **X** are taken into consideration.

In medical research, a frequently used method to estimate the conditional hazard function is the Cox proportional model (see [9]), which is defined as

$$\begin{array}{c} \hat{\lambda }\left(t|x\right)={\hat{\lambda }}_{0}\left(t\right)e^{{\hat{\beta }}^{T}x}={\hat{\lambda }}_{0}\left(t\right)e^{\sum_{i=1}^{p}{\hat{\beta }}_{i}x_{i}}, \end{array}$$

where ${\hat{\lambda }}_{0}\left(t\right)$ is a nonparametric estimate of baseline hazard function and $\hat{\beta }$ is the maximum-likelihood estimate of vector of parameters.

The advantage of the model consists in the fact that no distribution of the lifetime is assumed and that the parameters are easy to estimate and interpret using the hazard ratio. The Cox model only imposes the proportionality of the hazard rate, i.e., the hazard ratio is constant over time. However, this assumption can sometimes be too restrictive. Thus we use the kernel method proposed in [10] or [11], which is able to overcome this disadvantage. Both approaches are compared in the next section.

The kernel estimator of the conditional hazard function for observed times *Y*_*i*_, censoring indicators *δ*_*i*_ and covariates *X*_*i*_ is in the form

$$\begin{array}{c} \hat{\lambda }\left(t|x\right)=\frac{\frac{1}{h_{t}}\sum_{i=1}^{n}w_{i}\left(x\right)K\left(\frac{t-Y_{i}}{h_{t}}\right)\delta_{i}}{\sum_{i=1}^{n}w_{i}\left(x\right)W\left(\frac{Y_{i}-t}{h_{t}}\right)}, \end{array}$$

where *W*(⋅) is a kernel distribution function and *w*_*i*_(*x*) are the Nadaraya—Watson weights defined by

$$\begin{array}{c} w_{i}\left(x\right)=\frac{K\left(\frac{x-X_{i}}{h_{x}}\right)}{\sum_{j=1}^{n}K\left(\frac{x-X_{j}}{h_{x}}\right)},\;i=1,\cdots ,n. \end{array}$$

Two bandwidths determine the size of neighborhood in this conditional case—*h*_*x*_ in the direction of the covariate and *h*_*t*_ in the direction of time.

The kernel estimator of the conditional survival function is obtained using the relationship to the conditional hazard function:

$$\begin{array}{c} \hat{\bar{F}}\left(t|x\right)=e^{-\int_{0}^{t}\hat{\lambda }\left(u|x\right)du}. \end{array}$$

The Cox regression model is applied in two different ways. The first one is to compare it with the kernel estimate of hazard function conditional to age (or GTV). The second one is more complex, to study the association of risk with carefully chosen variables such as age and GTV, sex, diagnosis, KI, stereotactic methods and tumor location. These variables were included in the saturated Cox model from which the sub-optimal model was selected by step-wise elimination of non-significant variables.

It would be possible to evaluate the kernel estimate of hazard function for particular levels of categorical variables, however, we did not have a sufficient number of observations (data).

To test the hazard ratio of variables from Table 1 (univariate setting) we use two- and more- sample log-rank tests at a significance level *α* = 0.05. In all other situations (Cox regression model for age and GTV, sub-optimal Cox regression model) we used Wald tests at the same *α*.

All statistical analyses were performed using R software version 3.1.3 [12] and MATLAB version 2012b.

## Results

**Fig 1:**
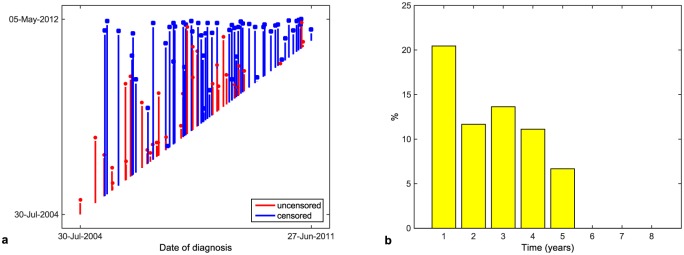
Patients’ times (a), death rate in separate years (b).

The period of time that a patient spends in a study measured from date of the diagnosis is referred to as a patient’s time. The times of patients for the given data set are presented in Fig 1a. The death rate in particular years from the date of diagnosis can be seen in Fig 1b.

**Fig 2:**
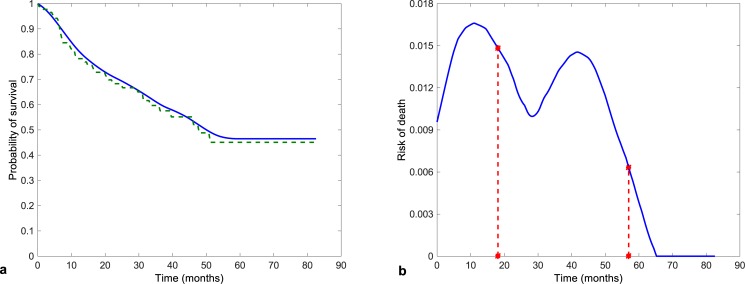
Estimates of the survival function (a) and kernel estimate of the hazard function (b) with points of greatest decrease.

The Kaplan—Meier estimate of the survival function (the dashed line) together with the kernel estimate (the solid line) is shown in Fig 2a and the kernel estimate of the hazard function is presented in Fig 2b. The median survival in our cohort of patients was 47.8 months. Three-year and five-year survival were estimated to be 57.5% and 45.2%.

The speed of decrease of the survival function is captured by the hazard function. The estimate of the hazard function has two peaks. The first one at about 10 months was due to death from high-grade glioma. The second one was probably caused by relapse of the other types of tumor. The descent of the survival function is the greatest at these times.

The estimate of the hazard function makes it possible to detect points of the most rapid change. These points (*θ*_1_ = 18.108 months, *θ*_2_ = 56.952 months) are marked in Fig 2b.

By univariate analysis, the survival of our patients differed significantly depending on the diagnosis, KI and stereotactic methods. The results are summarized in Table 3.

**Table 3:** Results of the univariate analysis—p-value (Statistically significant p-values are highlighted in bold.), hazard ratio and Wald 95% empirical confidence interval characterized by its lower and upper bounds (LB, resp. UB).

	p-value	HR	95% LB	95% UB
**Sex** (male vs female)	0.230	1.503	0.769	2.939
**Diagnosis**	≪**0.001**			
**Location** (supra- vs infratentorial)	0.110	2.283	0.805	6.473
**KI** (<80% vs ≥ 80%)	**0.047**	2.114	0.994	4.495
**Stereotactic methods** (SRT vs SRS)	**0.033**	2.713	1.047	7.027

A standard statistical test for comparing survival functions cannot be used for continuous characteristics. We could use the Cox model but we would need to assume an exponential dependence. The kernel estimations help us find out the influence of such characteristics.

**Fig 3:**
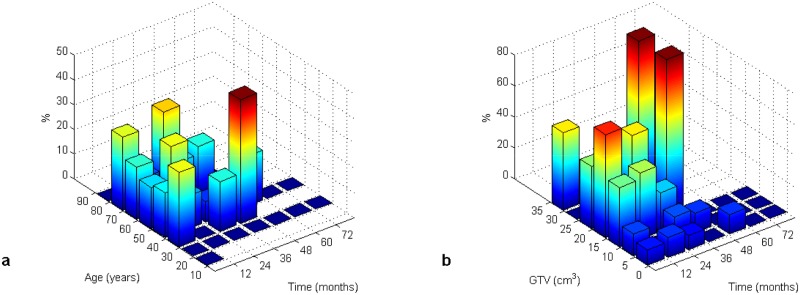
Death rate (in %) with age (a) and GTV (b) as covariates.

Fig 3 presents the death rate (in %) with age as a covariate and the death rate with the volume of tumor as a covariate, respectively. In these figures we can see the probability of death as an interaction of age (or GTV) and survival time intervals.

**Fig 4:**
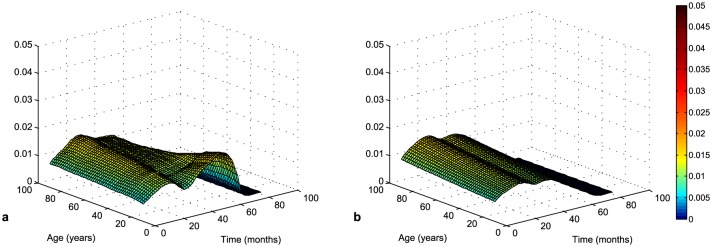
Kernel (a) and Cox (b, $\hat{\beta }=0.004$, p-value = 0.696) estimate of conditional hazard function with age as a covariate.

The kernel and the Cox estimates of the conditional hazard function with age as covariate are presented in Fig 4. The Cox model shows the risk of death does not change with age ($\hat{\beta }=0.004$, p-value = 0.698). On the contrary, the kernel estimate indicates that the highest risk is for younger patients with a survival time of about 50 months.

**Fig 5:**
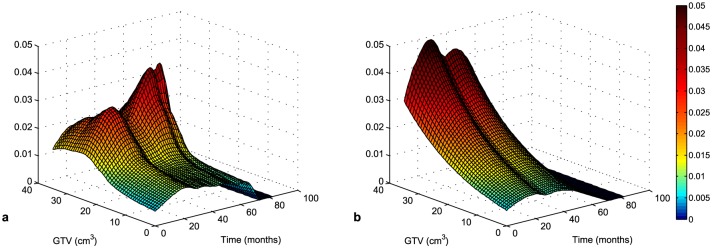
Kernel (a) and Cox (b, $\hat{\beta }=0.060$, p-value = 0.009) estimate of conditional hazard function with GTV as a covariate.

In Fig 5, both estimates captured the increase of risk with GTV, however, the kernel estimate can distinguish different slopes in different survival times. In the direction of time of survival, the shape of the hazard function is similar to the unconditional one in Fig 2b. Large tumors are more difficult to treat, therefore the patients with a greater GTV have a higher risk of death. The patients with LG glioma have a less aggressive tumor and therefore their survival time is longer.

We could divide patients into groups where they would have a similar risk of death. This partition would simplify the interpretation of results. The kernel estimate can be helpful in determining the thresholds of characteristics for dividing into groups. A good threshold for GTV can be for example 20 cm^3^ according to Fig 5. The risk of death is low for patients with a GTV less than 20 cm^3^ and high for patient with a greater GTV, while dependence on time is similar.

It is evident that due to proportionality the Cox model cannot reflect the influence of covariates. If data matched the Cox model, both methods, kernel estimation and Cox model, would have similar estimates. We can see it in the case of dependence on age in Fig 4. In such situations, it is better to use the Cox model due to easier interpretation. In the case of dependence on GTV, the violation of assumptions is more pronounced. The hazard ratio is constant over time but the dependence on GTV is not exponential.

**Fig 6:**
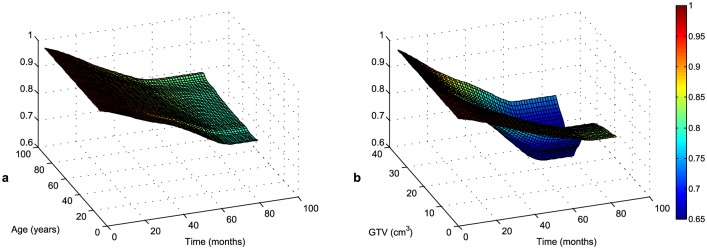
Kernel estimates of conditional survival function with age (a) and GTV (b) as a covariate. (Note that probability of survival is displayed from value 0.6 due to visibility.)

As in the unconditional case, we can present the survival function as dependent on a covariate. Due to the relationship between the survival and hazard functions, Fig 6 provides an alternative view on a patient’s survival.

For simplicity, we assume exponential dependence of these characteristics for the multivariate model in view of the fact that there is not too much difference between kernel and Cox estimates and that the exponential dependence condition is not too violated.

As a result of the Cox regression model (Table 4), an increase in age of about one year is followed by a 1.075-fold increase in risk of death, and an increase in GTV by one cm^3^ is followed by 1.084-fold increase in risk of death. As far as the diagnoses is concerned, the hazard ratio of LG glioma to meningioma is equal to 338.311, the hazard ratio of HG glioma to meningioma 177.544 and the hazard ratio of other diagnoses to meningioma 10.581. The hazard ratio of KI smaller than 80 to KI greater than or equal to 80 is equal to 2.720. From the whole set of interactions, the most important are sex with diagnosis and sex with tumor location. The hazard ratio of females with meningioma to males with LG glioma is equal to 1/0.017 = 60.123, the hazard ratio of females with meningioma to males with HG glioma 1/0.131 = 7.605 and the hazard ratio of males with supratentorial location to females with infratentorial location 116.881. In contrast to the univariate analysis (Table 3), the hazard ratio of stereotactic methods is not significantly different from one due to the influence of other characteristics in the Cox model (Table 4) and it is therefore not a part of the sub-optimal model.

**Table 4:** Results of the Cox regression model—regression coefficient $\hat{\beta }$, standard error se $\hat{\beta }$, *Z*-statistics, p-value (Statistically significant p-values are highlighted in bold.), hazard ratio $\exp(\hat{\beta })$ and Wald 95% empirical confidence interval characterized by its lower and upper bounds (LB, resp. UB).

	$\hat{\beta }$	se $\hat{\beta }$	*Z*-stat	p-value	$\exp(\hat{\beta })$	95% LB	95% UB
**Age (years)**	0.072	0.018	4.048	**≪ 0.001**	1.075	1.038	1.113
**GTV (cm^3^)**	0.081	0.026	3.116	**0.002**	1.084	1.030	1.140
**Sex**							
male	−2.438	1.917	−1.272	0.203	0.087	0.002	3.737
**Diagnosis**							
LG glioma	5.824	1.298	4.486	**≪ 0.001**	338.311	26.562	4308.994
HG glioma	5.179	0.954	5.432	**≪ 0.001**	177.544	27.394	1150.701
other	2.359	1.075	2.194	**0.028**	10.581	1.286	87.021
**Karnofsky index**							
KI<80	1.001	0.464	2.155	**0.031**	2.720	1.095	6.758
**Location**							
supratentorial	−1.521	1.027	−1.480	0.139	0.219	0.029	1.637
**Sex:Diagnosis**							
male:LG glioma	−4.096	1.327	−3.087	**0.002**	0.017	0.001	0.224
male:HG glioma	−2.029	0.929	−2.185	**0.029**	0.131	0.021	0.811
male:other	−0.493	1.615	−0.305	0.760	0.611	0.026	14.465
**Sex:Location**							
sex:supratentorial	4.761	1.902	2.503	**0.012**	116.881	2.808	4864.614

## Discussion

Stereotactic radiation methods have their place in the treatment of brain lesions. In the case of primary brain tumors, they are used primarily to treat a recurrent disease, the tumor bed after incomplete extirpation, or in the localization of tumors in eloquent areas. Meningiomas are the most common diagnosis for their use and make up 15–25% of intracranial tumors. Clinical symptoms depend on their size and location. There are many studies dealing with the treatment methods of meningioma using SRT or SRS with the use of X knife— [13], [14], [15] or [16] where there is an analysis of several published studies. Metellus and others [17] looked for the differences between SRT and SRS in the treatment of meningioma.

The group of high-grade gliomas belongs to the most aggressive tumors. Their prognosis, despite of all advances in medicine, is poor. In the treatment strategy, radiotherapy is applied primarily as an adjuvant method. The primary method of treatment is surgery. At relapse, radiotherapy may be one of the possible alternatives for salvage therapy. Due to the high dose already applied in the postoperative period, the possibilities of conformal radiotherapy are limited. It may be replaced by stereotactic radiotherapy, however, the results are not good [18]. There are again some studies dealing with glioblastoma— [19], [20], or [21].

We assessed the results of several authors who dealt with monitoring survival, progression or remission for patients with a primary brain tumor. From a perspective of statistical evaluation, the mentioned studies are on different levels. Most of these authors (e.g. [13], [14], [19]) used only descriptive statistics or the Kaplan—Meier estimate of the survival function and univariate analysis with a log-rank test and a Gehan—Wilcoxon test. Only some authors proposed the multivariate Cox model ([14] or [15]). However, none of the authors used the hazard function which offers a better examination of the risk of death. The kernel estimate of the conditional hazard function can be useful for verifying assumptions of the Cox model, for finding thresholds of continuous variables or for replacing the Cox model where assumptions are violated.

Rodrigues and others [22] used an alternative approach to statistical analysis. In addition to the classical method (Kaplan—Meier estimate or univariate and multivariate Cox model), they used time-dependent ROC curves. This analysis was introduced by Heagerty and others [23]. ROC curves are a method for displaying the sensitivity and specificity of continuous diagnostic markers. They can also be used in survival analysis. However, the vital status is a time-dependent variable and we obtain several ROC curves dependent on time. Based on ROC analysis, the significance or the threshold of a diagnostic marker can be determined. The optimal threshold can be identified using some optimality criterion (e.g. [24]). Note that several thresholds might be equally optimal. Moreover, we can obtain various thresholds for different time points. In this respect, the conditional hazard function is more flexible and offers a complex perspective. Based on the conditional hazard function graph, we can consider the suitability of using a threshold, to assess its dependence on time or determine more than one threshold. Both ROC analysis and the conditional hazard function provide a different view on the influence of the marker on death. ROC curves are based on probability along a time interval. On the other hand, the conditional hazard function provides an immediate risk of death at a specific time.

### Summary

In medical research, classical methods such as the Kaplan—Meier estimate of the survival function and the Cox regression model are frequently used. However, we propose a different approach to the analysis of survival data though hazard and conditional hazard functions. Both classical and proposed methods give a more complex basis for the interpretation of obtained results.

The achieved results can be summarized as follows:

The Cox model shows a higher risk of death for an increasing volume in tumors.

The kernel estimate indicates the highest risk for a greater tumor volume in a period of about 50 months from the beginning of therapy.

The kernel estimate is able to capture any changes in the hazard function in the direction of the covariate and time, which cannot be described by the exponential nature of the Cox regression model.

The methods presented provide the possibility of evaluating continuous risk variables and according to the results offer risk-prone patients a more aggressive treatment. To conclude especially careful surveillance of younger patients can be recommended.

The kernel estimate of the conditional hazard function can be useful for verifying the assumptions of the Cox model and for finding thresholds of continuous variables. These thresholds divide patients to two or more groups with similar risk of death. In case of our data, a suitable threshold for GTV can be 20 cm^3^, i.e. the patients with GTV smaller than 20 cm^3^ have lower risk of death than the patients with GTV greater or equal than 20 cm^3^. Finally, proposed kernel methods are able to estimate different shapes of the hazard function without any constrains and smooth them out. This flexibility can be used not only in brain tumor research but also in other fields applying methods of survival analysis. A suitable threshold for GTV can be 20 cm^3^.

## Supporting Information

S1 Dataset Data used in analyses. (XLSX)
